# Validation of liquid biopsy for ESR1-mutation analysis in hormone-sensitive breast cancer: a pooled meta-analysis

**DOI:** 10.3389/fonc.2023.1221773

**Published:** 2023-08-22

**Authors:** Omar Najim, Konstantinos Papadimitriou, Glenn Broeckx, Manon Huizing, Wiebren Tjalma

**Affiliations:** ^1^ Multidisciplinary Breast Clinic Antwerp University Hospital, University of Antwerp, Edegem, Belgium; ^2^ Faculty of Medicine, University of Antwerp, Edegem, Belgium; ^3^ Department of Medical Oncology, Antwerp University Hospital, Edegem, Belgium; ^4^ Department of Pathology, Antwerp University Hospital, Edegem, Belgium; ^5^ Biobank, Antwerp University Hospital, Edegem, Belgium; ^6^ Unit of Gynecologic Oncology, Department of Obstetrics and Gynecology, Antwerp University Hospital, University of Antwerp, Edegem, Belgium

**Keywords:** ESR1, liquid biopsy, next-generation sequencing, digital PCR, metastasized breast cancer

## Abstract

Several retrospective and prospective studies have shown that genomic alterations in Estrogen-receptor one (ESR1) can be characterized not only in tissue samples but also by sequencing circulating tumor DNA (ctDNA) in liquid biopsy. Therefore, liquid biopsy is a potential noninvasive surrogate for tissue biopsy. This meta-analysis was designed to compare the prevalence of ESR 1 mutation detected with liquid biopsy and tissue biopsy. A pooled meta-analysis of studies published between 1 January 2007 and 1 March 2021 was conducted regarding the methodologies used for ESR1 mutation analysis. Liquid biopsy is a valid, inexpensive, and attractive noninvasive alternative to tumor biopsies for the identification of ESR1 mutations. Liquid biopsy for ESR 1 analysis would facilitate regular testing, allowing monitoring of the sensitivity to ET and guiding treatment strategies.

## Introduction

Our understanding of cancer biology using minimally invasive techniques to collect circulating tumor DNA (ctDNA) from body fluids is rapidly evolving. The fragmented DNA segments found in blood samples of cancer patients could be used to validate the presence of tumor specific mutations (1–3).

Breast tumors commonly express hormone receptors (HR), including the estrogen receptor (ER) and/or progesterone receptor (PR) (4). Endocrine therapy (ET), which targets the ER pathway, is a major treatment modality for HR-positive cancers. At diagnosis, ER positivity is a favorable prognostic factor for breast cancer (BC). However, this positive prognostic effect degrades over time (5). Resistance to ET is considered an important step in the natural evolution of HR-positive BC and is related to a higher risk of recurrence and increased mortality (6). In the last decade, several clinical trials have assessed the incidence of ESR1 mutations in BC based on liquid biopsies. This knowledge is likely to encompass important information on the development of resistance to ET in real time, and is eventually applied for patient/treatment selection and monitoring of ET efficacy (7, 8).

Currently, the detection and molecular characterization of ctDNA represents one of the most active fields of translational cancer research. The recent development of NGS has expanded the monitoring of ctDNA with a range of diagnostic clinical applications. However, there are several limitations, including difficulties in interpreting novel or rare mutations and cost issues (9). On the other hand, the newly developed digital polymerase chain reaction (dPCR) has the potential to detect rare mutants, in which a variant of a single-nucleotide polymorphism is predominantly present among wild-type sequences (10). Droplet digital polymerase chain reaction (ddPCR), which can perform thousands of PCRs on a nanoliter scale simultaneously, would be an attractive method for massive parallel sequencing to identify the significance of low-frequency rare mutations. ddPCR is the most appropriate method for detecting known hotspot mutations, but is not the most appropriate approach for detecting unknown and ‘not-targeted’ mutations (11). Compared with singleplex reactions, multiplexing ddPCR not only increases the number of targets measured in a single reaction but also reduces the amount of clinical material required to analyze multiple single-nucleotide polymorphisms by measuring >1 target in a single reaction (12).

In BC, as in other solid tumors, the genomic alterations found within a given tumor biopsy may differ depending on the region sampled, as between the primary tumor and metastatic deposits, and even between different metastatic deposits (13). Genomic analyses of BC have provided direct evidence of spatial and temporal intratumoral heterogeneity (14, 15). Currently, clinical and therapeutic decisions are based on individual tissue biopsies that may not be representative of the entire tumor burden or on real-time assessments of the tumor genotype (16). This practical limitation could be overcome by the use of liquid biopsies, which represent a promising technique for decoding tumor heterogeneity.

In this review, we compare the prevalence ESR1 mutations for female patients with ER+ recurrent/metastasized BC pretreated with ET as detected by liquid biopsy versus standard tissue biopsy. This review discusses and summarizes the techniques of DNA sequencing, including ddPCR and NGS, which are used by several laboratories to address the potential clinical needs of ESR1 mutation-specific BC. A thorough understanding of these applications may provide useful information for ESR1 testing, ensure reliable test results for use in clinical practice, and eventually advance personalized therapeutic strategies. Aromatase Inhibitors (AIs) reduce circulating estrogen by inhibiting estrogen synthesis in peripheral tissues by 90% or more, but do not affect estrogen production in the ovaries. ESR1 mutations allow ERα to be activated in the absence of estradiol, eliminating AIs activity and making ESR1 a potential predictive factor.

## Materials and methods

A literature search was conducted using two databases: PubMed and Thomson Reuters Web of Science. The following search terms were used: [(‘liquid biopsy’ OR ‘tissue sample’) AND (‘ESR1 mutation’ OR ‘ESR mutation’) AND (‘next generation sequencing’ OR ‘ digital PCR) AND (‘breast cancer’)]. The reviewers performed the procedure of study selection by: (1) assessment of each clinical trial independently in an unblended standardized manner; (2) duplicates were removed afterwards; (3) only full-text English articles were included; (4) after the independent screening, all the results were compared and the articles with conflict were discussed until agreement was established; (5) the article should refer to an interventional trial; reviews, lectures and book sections were excluded; and (6) the final decision for study selection of the remaining articles were treated separately; studies that did not meet the inclusion criteria or did not contain useful information for this systematic review were excluded after consensus.

The included articles were published between 1 January 2007 and 1 March 2021. The extracted data included the type of clinical trial (RCT or non-RCT), characteristics of the study population, number of participants, exclusion of primary disease, nature of biopsy samples (plasma or tissue), and method of mutation analysis (ddPCR or NGS). The selected patients met the following inclusion criteria: 1. Female aged >18 years, 2. ER+ breast cancer cells pre-treated with endocrine therapy, and 3. Disease recurrence and metastases. Patients with primary breast cancer were excluded from this study. Overall incidence of ESR1 mutation was assessed using a meta analysis for proportions. Because of high diversity in type of studies, patients and therapies, a random effects model is used. Heterogeneity is judged by forest plot, Cochran Q and I-squared. Results are presented in a forest plot for proportions. Incidence of ESR1 mutation was compared between plasma versus tissue samples and between ddPCR versus NGS. Subgroup differences are evaluated by the between subgroups heterogeneity statistic in the random effects meta-analysis. P-values were considered statistically significant if it was < 0,05.

## Results

**Figure 1:**
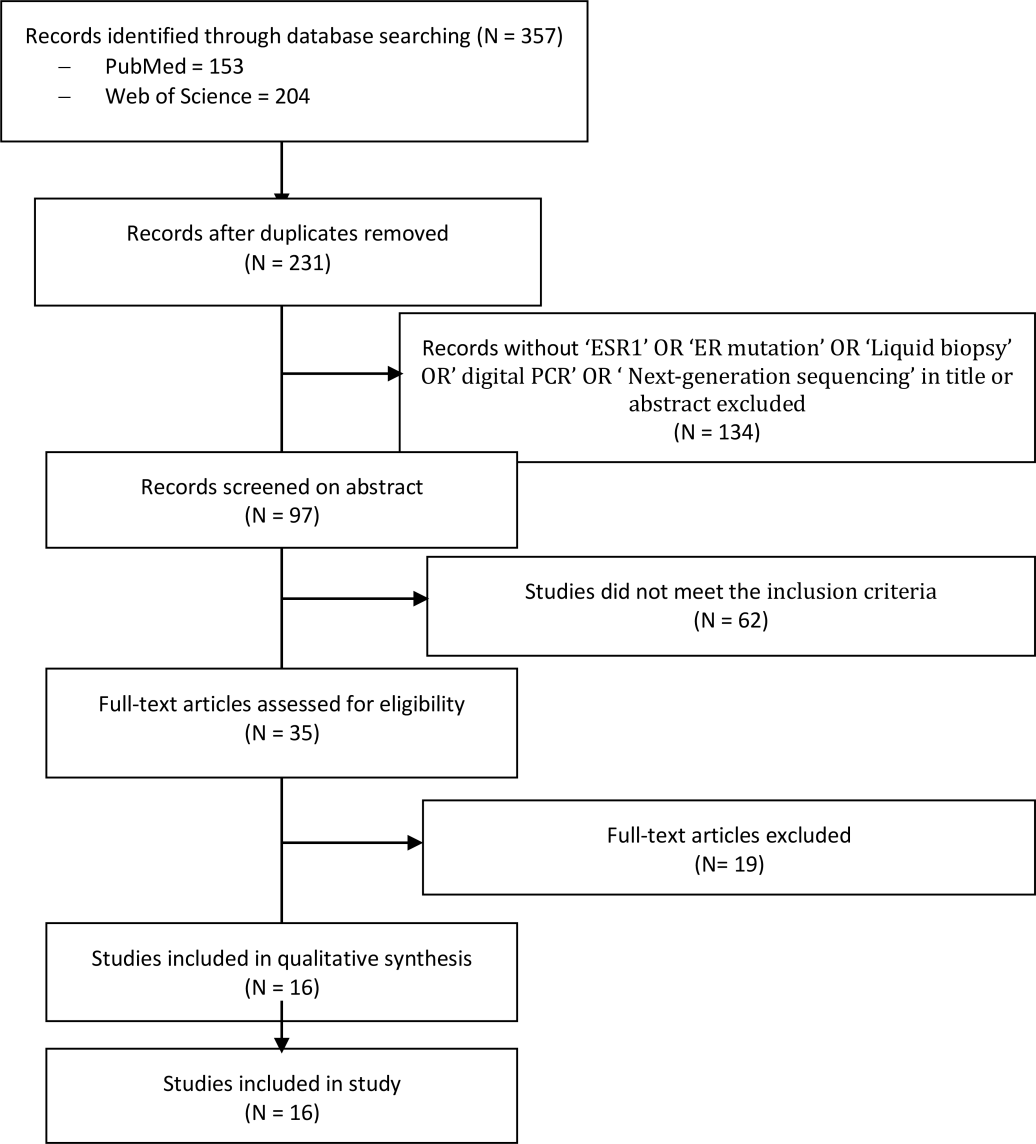
PRISMA flow diagram and the process of data selection. Selection of studies was performed using predefined data fields, taking study quality indicators into consideration. Eligibility criteria included terms with ‘ESR1’, ‘ESR mutation’ and ‘liquid biopsy’ or ‘tissue sample’, and/or ‘next generation sequencing’ and/or ‘ddPCR’ in the abstract or title by using the endnote library search option.

A literature search fulfilling the previously explained search criteria and taking place in the proposed time interval resulted in a collection of 153 articles in PubMed and 204 articles in Web of Science. A total of 231 articles were evaluated after excluding duplicates. Articles that did not meet the inclusion criteria or that did not contain useful information for this systematic review, were discarded after consensus. Thereafter, 16 articles, four multicenter double-blinded RCTs, and 12 cohort trials were obtained for this meta-analysis (**Figure 1**).

From the reviewed studies, we included 2,744 pooled tissues and plasma samples for this analysis. Plasma samples were used in 57.1% (1,568/2,744) of the study population, tissue samples in 37.7% (1,033/2,744), and tissue-plasma pairs in 5.2% (143/2,744). Tissue samples were obtained from loco regional or distant metastatic sites in four and six studies, respectively. Both archived and recent plasma samples were used for ESR1 analysis in four and two studies, respectively. ESR1 analysis was performed using ddPCR in 61.3% (1,684/2,744) of the study population and NGS in 38.7% (1,060/2,744).

**Figure 2:**
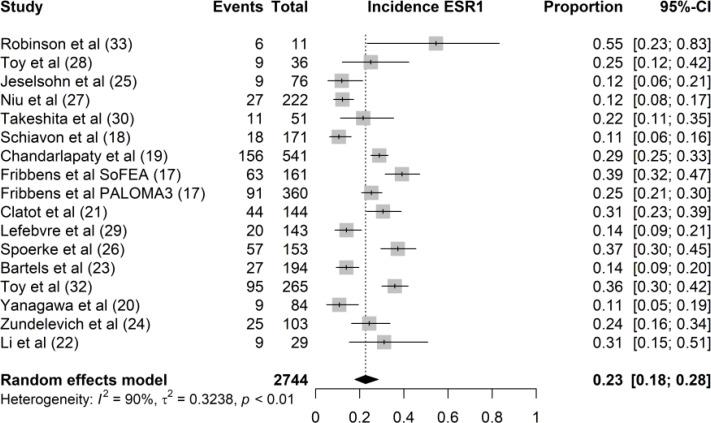
Forest plot of the overall incidence rate of ESR1 mutation. The proportion of ESR1 mutation per study is displayed with a grey box, with the 95%-CI visualized by horizontal lines. The overall frequency of ESR1 mutations was 0.23 (95%-CI: 0.18 0.28), as indicated by the black diamond at the bottom of the forest plot.

Of the 2,744 samples pooled for this study, the overall incidence of ESR1 mutation is 23% (95 CI 18%–28%) (**Figure 2**). However, the different studies demonstrated a considerable variability in the prevalence of ESR1 mutations ranged from 11% in Schiavon et al. (17) and Yanagawa et al. (18) to 55% in Robinson et al. (19) The wide range in incidence rate of ESR1 mutation could be attributed to heterogeneity in the study populations.

**Figure 3:**
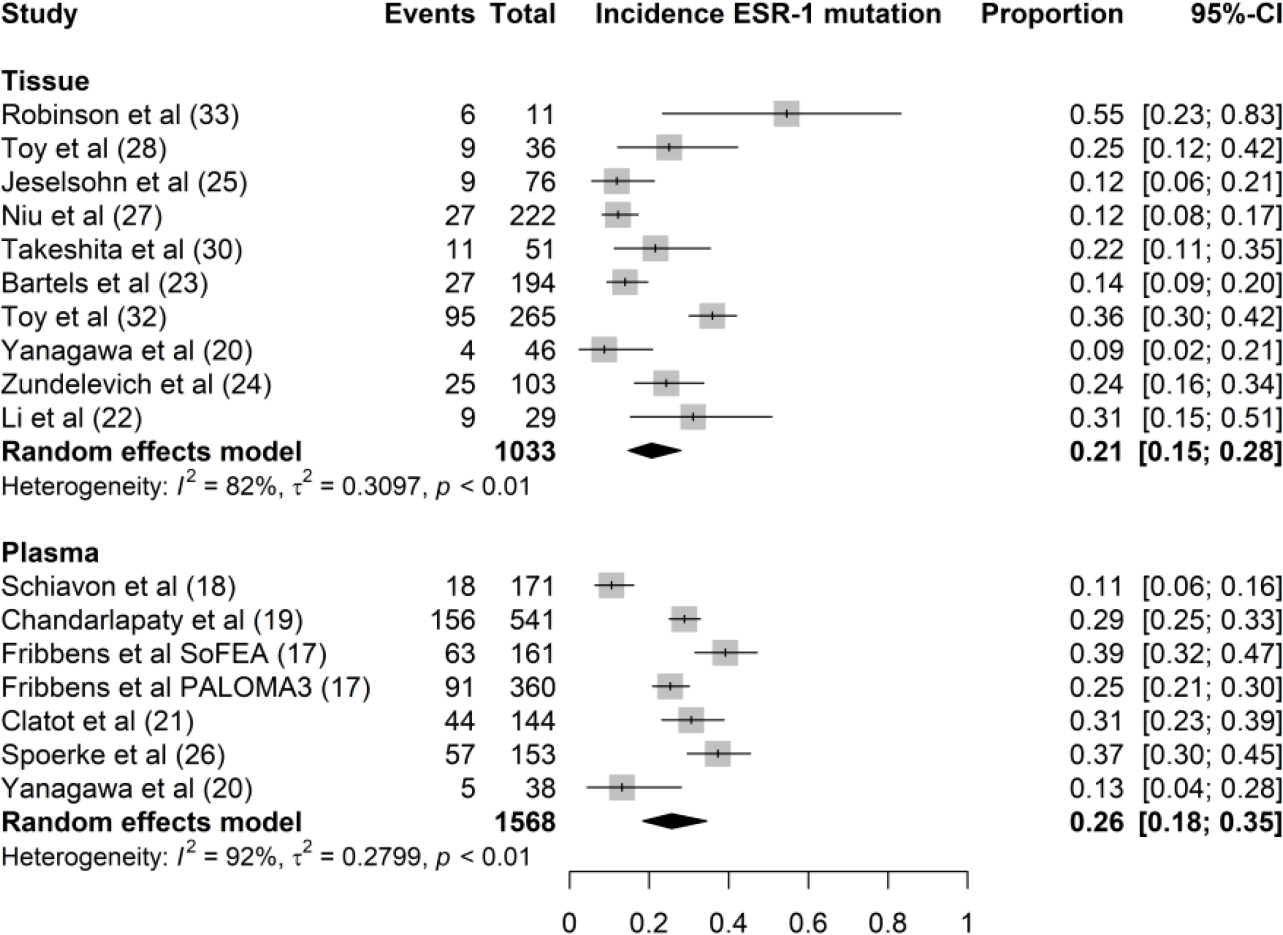
Forest plot of the comparison of ESR1 mutation in tissue versus plasma samples. Grey boxes indicate the proportion of ESR1 mutations in each study, with a horizontal line representing the 95% CI. Overall proportion and 95% CI in tissue and plasma subgroup is displayed with a black diamond. We found no significant difference in ESR1 mutation incidence between plasma and tissue samples (P=0.34).

In the articles under review, nine studies used tissue biopsy while five studies used plasma biopsy. In a trial by Yanagawa et al. (18), whole-exon sequencing of the ESR1 gene was performed separately in tissue and plasma samples. In 15 of the 16 studies included, the incidence rates of ESR1 mutations in plasma and tissue samples were 26% (95% CI, 18%–35%) and 21% (95% CI, 15%–28%), respectively (**Figure 3**). We found no significant difference in ESR1 mutation incidence between plasma and tissue samples (P = 0.34). The samples from Lefebvre et al. (20) were excluded from the comparative analysis between liquid and tissue biopsies because ESR1 sequencing was performed in tissue-blood pairs. In this study, the mutational profiles of 143 tissue-blood pairs from patients with hormone receptor-positive (HR+) metastatic BC were analyzed. Twelve genes (TP53, PIK3CA, GATA3, ESR1, MAP3K1, CDH1, AKT1, MAP2K4, RB1, PTEN, CBFB, and CDKN2A) were significantly mutated in MBC. This study concluded that ESR1 mutation was the most frequent mutation in the HR+ MBC subgroup (n = 143). In total, 22 mutations were identified in 20 of 143 patients with HR+/HER2− BC (14%). Li et al. demonstrated that ESR1 mutations could be detected by serial monitoring of ctDNA. In this study, mutation profiles, including ESR1, were highly concordant between plasma and paired tissue samples from 45 patients with MBC (20).

**Figure 4:**
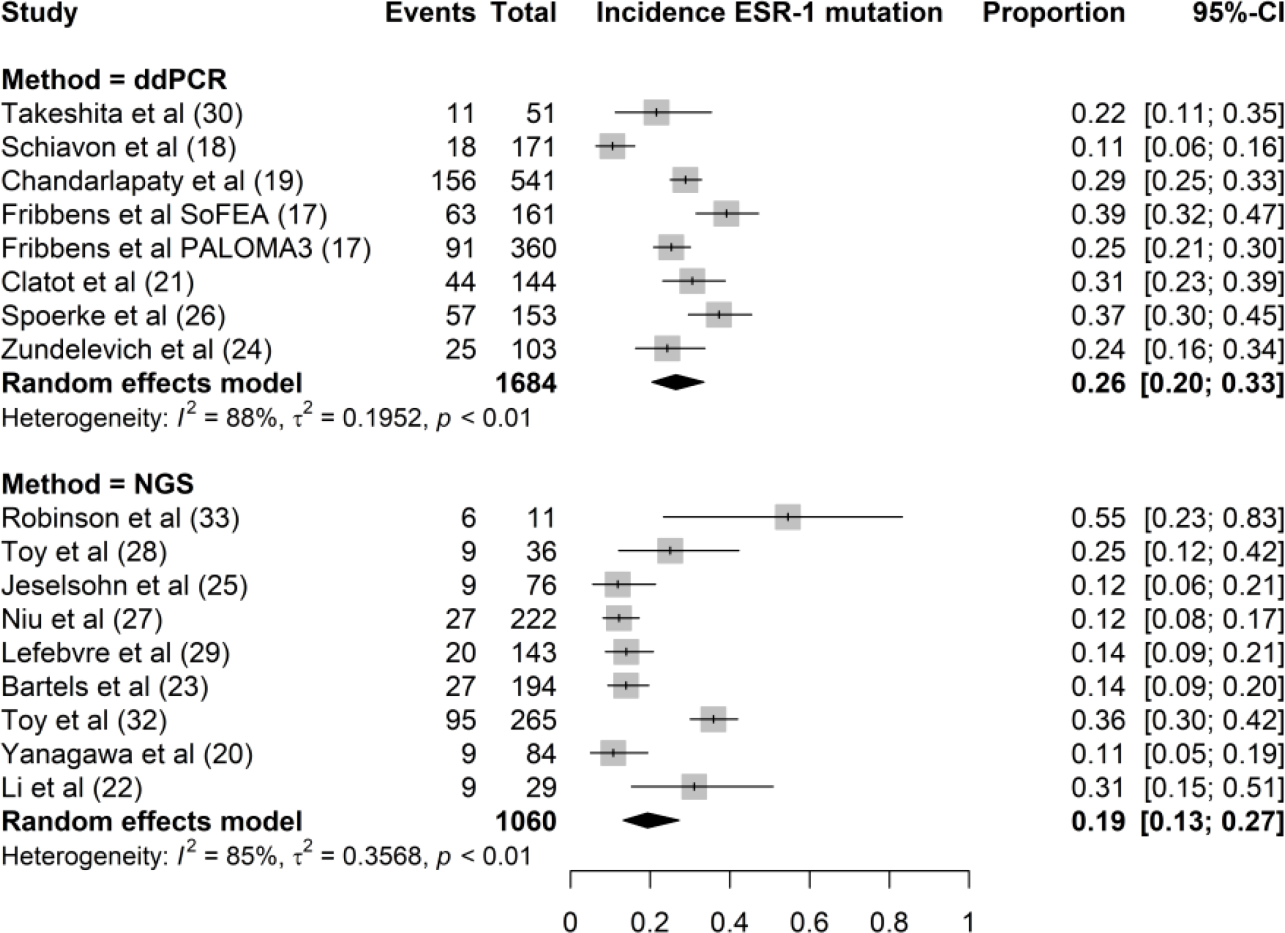
Forest plot of the comparison of the proportion of ESR1 mutation using NGS versus ddPCR techniques. Grey boxes indicate the proportion of ESR1 mutations in each study, with a horizontal line representing the 95% CI. Overall proportion and 95% CI in NGS and ddPCR subgroup is displayed with a black diamond. We found no significant difference in ESR1 mutation incidence between the two techniques (P=0.15).

Both ddPCR and NGS were used to determine ESR1 mutations in the tissue and plasma samples. ddPCR was used in seven studies and NGS was used in nine studies. ddPCR is the standard method for ESR1 testing in liquid biopsies, except in the study by Yanagawa et al. NGS was used to analyze both tissue and plasma samples. However, both NGS and ddPCR have been used for ESR1 testing of tissue biopsies. The incidence rates of ESR1 mutations using ddPCR and NGS were 26% (95% CI, 20%–33%) and 19% (95% CI, 13%–27%), respectively (**Figure 4**). We found no significant difference in ESR1 mutation incidence between ddPCR and NGS techniques (P = 0.15).

All studies on both plasma and tissue samples have described their methodology regarding collection, processing, and analysis in a more or less complete manner, despite some missing pre-analytical aspects (17–19, 21–32). Of the six studies researching plasma samples, only one used NGS (18), while the other studies used ddPCR (17, 21–24). Two of the 10 studies used ddPCR (29, 32), while eight other studies used NGS (18, 19, 25–28, 30, 31). Remarkably, all studies performing ddPCR, whether on tissue samples or plasma samples, used the same platform (Bio-Rad QX200 ddPCR system) and more or less the same pre-analytical and DNA-quantification steps; however, the hotspot mutation panel might differ according to the respective study (17, 21–24, 29, 32). In contrast, many different NGS platforms are used, with many different library preparation kits and quantification tools. Some of the NGS platforms used are the Illumina HiSeq 2000 series and the Ion Torrent platform (18, 19, 25–27, 30, 31). Additionally and important to note, genomic profiling was performed by Foundation Medicine on the Foundation One platform in one study on tissue samples (28). As this study did not aim to investigate the different aspects of the ESR1 analysis methodology, we will not go into detail in the different preanalytical, DNA-quantification, and mutation analysis steps. Nonetheless, these data can be found in the schematic overview of the available preanalytical and analytical parameters provided in **Tables 1**, **2**. We previously published in an earlier review a detailed ESR1 specific mutational profile analysis, including D538G, Y537S, and Y537N as the most prevalent mutations (33).

**Table 1:** Overview of the collection, processing and ESR1 mutation analysis in all studies concerning the analysis of plasma samples.

Study	Collection	TTP	Centrifugation		Volume cleared plasma	Storage	DNA extraction kit	DNA quantification				Analysis	Comments
			Speed	Time				Method	Reference gene	Mass	Concentration		
**Fribbens et al. 2016** (17) **SoFEA trial**	EDTA tubes	0-9 days	1600 g	20 minutes	/	-80°C	QIAamp Circulating Nucleic Acid Kit (Qiagen, Hilden, Germany)	ddPCR (Bio-Rad QX200 system)	RNase P		10²-10^7^copies/mL	Multiplex ddPCR and characterization on uniplex ddPCR (Bio-Rad QX200 system)	Multiplex 1: c.1138G.C(E380Q), c.1607T.G(L536R), c.1610A.G(Y537C), c.1613A.G(D538G) Multiplex 2: c.1387T.C(S463P), c.1609T.A(Y537N), c.1610A.C(Y537S)
**Fribbens et al.** **2016** (17) **PALOMA3 study**	EDTA tubes	0-30 minutes	1500-2000 g	10 minutes	/	-80°C	QIAamp Circulating Nucleic Acid Kit (Qiagen, Hilden, Germany)	ddPCR (Bio-Rad QX200 system)	RNase P		10²-10^7^copies/mL	Multiplex ddPCR and characterization on uniplex ddPCR (Bio-Rad QX200 system)	Multiplex 1: c.1138G.C(E380Q), c.1607T.G(L536R), c.1610A.G(Y537C), c.1613A.G(D538G) Multiplex 2: c.1387T.C(S463P), c.1609T.A(Y537N), c.1610A.C(Y537S)
**Schiavon et al.** **2015** (18)	EDTA tubes	0-2 hours	1600 g	20 minutes	/	-20°C	QIAamp Circulating Nucleic Acid Kit (Qiagen, Hilden, Germany)	ddPCR (Bio-Rad QX200 system)	RNase P			Multiplex ddPCR and characterization on uniplex ddPCR (Bio-Rad QX200 system)	Multiplex 1: c.1607T.G(L536R), c.1610A.G(Y537C), c.1609T.A(Y537N) Multiplex 2: c.1610A.C(Y537S) c.1613A.G(D538G)
**Chandarlapaty et al.** **2016** (19)	EDTA tubes	0-30 minutes	1100-1300 g	/	0.3-3.3 mL (median 1.8 mL)	-70°C	QIAamp Circulating Nucleic Acid Kit (Qiagen, Hilden, Germany)	qPCR (KAPA Human Genomic DNA Quantification and QC kit)	/			Uniplex ddPCR (Bio-Rad QX200 system)	Uniplex: c.1610A.C(Y537S) c.1613A.G(D538G)
**Yanagawa et al.** **2017** (20)	/	/	3000 g	10 minutes	/	-80°C	QIAamp Circulating Nucleic Acid Kit (Qiagen, Hilden, Germany)					NGS (Thermo Fisher Ion Torrent PGM)	Primer design: Ion Ampliseq^TM^ Custom DNA Panels Library preparation: Ion Ampliseq Library Kit 2.0 MAF cut off 3.0%
**Clatot et al.** **2016** (21)	Heparinized tubes	0-2 hours	2000 g	10 min		4-20°C*	QIAamp Circulating Nucleic Acid Kit (Qiagen, Hilden, Germany)	Fluorometry (Invitrogen Quanti-IT^TM^ PicoGreen^®^ dsDNA Assay Kit)			200-2000 copies/mL	Multiplex ddPCR (Bio-Rad QX200 system)	Multiplex: c.1609T.A(Y537N), c.1610A.C(Y537S), c.1610A.G(Y537C), c.1613A.G(D538G)
**Spoerke et al.** **2016** (26)	EDTA	0-1 hour	820 g 16000 g	10 min 10 min		-80°C		qPCR (LINE-1) quantitative real-time PCR assay)		3-1500 ng		ddPCR OncoBEAM BC1 BEAMing Digital PCR panel	Panel: c.1138G.C(E380Q), c.1387T.C(S463P), c.1604C.A(P535H), c.1607T.A(L536H), c.1607T.C(L536P), c.1607_1608delTCinsAG(L536Q), c.1607T.G(L536R), c.1610A.G(Y537C) c.1609T.A(Y537N), c.1610A.C(Y537S), c.1613A.G(D538G)

TTP, time to preparation; EDTA. ddPCR, droplet digital polymerase chain reaction; qPCR, quantifying polymerase chain reaction; NGS, next generation sequencing; MAF, mutant allelic frequency.

**Table 2:** Overview of the collection, processing and ESR1 mutation analysis in all studies concerning the analysis of Tissue samples.

Study	Biopsy site	Preparation			DNA extraction	DNA Quantification	Analysis					Comments
		Medium	Slides				Type	Panel primer design	Library preparation	Quantification libraries	Instrument	
			Number	Thickness								
**Bartels et al.** **2018** (23)	Bone marrow	FFPE	2-6 slides	10µm	Maxwell RSC DNA FFPE Kit Maxwell RSC instrument	Fluorometry Qubit 2.0 Fluorometer dsDNA high sensitivity Assay Kit	NGS	Ion Ampliseq Designer	Ion Ampliseq Library kit 2.0	qPCR Ion Library Quantification Kit	Ion PGM Hi-Q Kit v2	
**Jeselsohn et al.** **2014** (25)	Primary site Metastatic sites	FFPE	/	40µm	Maxwell 16 FFPE Plus LEV DNA Purification Kit	Fluorometry PicoGreen fluorescence assay	NGS	/	RNA based baits hybridization	/	Illumina HiSeq2000	
**Li et al.** **2020** (22)	Metastatic sites Liquor	FFPE	/	/	/	Fluorometry PicoGreen fluorescence assay	NGS	/	KAPA Hyper DNA Library Prep Kit	/	Illumina HiSeq	
**Niu et al.** **2015** (27)	Primary site Metastatic site	/	/	/	/	/	NGS	/	/	/	/	Genomic profiling by Foundation Medicine on Foundation One platform
**Yanagawa et al.** **2017** (20)	Recurrent site Metastatic site	FFPE	3	10µm	QIAamp DNA FFPE Tissue Kit	/	NGS	Ion AmpliSeq Custom DNA Panels	Ion Ampliseq Library kit 2.0	/	Ion Torrent PGM	
**Robinson et al.** **2013** (33)	/	/	/	/	/	/	NGS	/	/	/	/	
**Takeshita et al.** **2015** (30)	Primary site Metastatic site	FFPE	/	/	AllPrep DNA/RNA Mini Kit PicoPure DNA Extraction Kit	Spectrophotometry NanoDrop 2000 Spectrometer	ddPCR	NA	NA	NA	Bio-Rad QX200 Droplet Digital PCR System	Uniplex ddPCR: c.1610A.C(Y537S), c.1609T.A(Y537N), c.1610A.G(Y537C), c.1613A.G(D538G)
**Toy et al.** **2013** (28)	Primary site Metastatic site	FFPE Fresh frozen	/	/	QuickGeneTM DNA tissue Kit	Fluorometry Nanodrop Fluorospectrometer	NGS	Agilent SureSelect Nimblegen SeqCap	Illumina TruSeq NEBNext DNA Library Prep Kit	Nimblegen SeqCap	Illumina HiSeq 2000	
**Toy et al.** **2017** (32)	Metastatic site	FFPE	15-20	10µm	QIAamp DNA Micro Kit	/	NGS	/	Nimblegen SeqCap	Nimblegen SeqCap	Illumina HiSeq 2500	
**Zundelevich et al.** **2020** (24)	Primary site Metastatic site	FFPE	1-10	10µm	All Prep DNA/RNA FFPE Kit	Fluorometry Qubit 2.0 Fluorometer dsDNA high sensitivity Assay kit	ddPCR	NA	NA	NA	Bio-Rad QX100 Droplet Digital PCR System Bio-Rad QX200 Droplet Digital PCR System	Uniplex ddPCR: c.1613A.G(D538G), c.1607T.G(L536R), c.1610A.C(Y537S), c.1609T.A(Y537N), c.1610A.G(Y537C)
**Schiavon et al.** **2015** (18)	Recurrent site Metastatic site	FFPE	4-8	4µm	QIAamp DNA FFPE Tissue Kit All Prep DNA/RNA FFPE Kit	ddPCR Bio-Rad QX200 Digital Droplet PCR Reference gene: RNase P	ddPCR	NA	NA	NA	Bio-Rad QX200 Droplet Digital PCR System	Multiplex ddPCR 1: c.1607T.G(L536R), c.1610A.G(Y537C), c.1609T.A(Y537N) Multiplex ddPCR 2: c.1610A.C(Y537S) c.1613A.G(D538G)

TTP, time to preparation;. ddPCR, droplet digital polymerase chain reaction; qPCR, quantifying polymerase chain reaction; NGS, next generation sequencing; NA, not applicable.

## Discussion

A growing body of clinical trials on ER+ BC strongly supports the use of ESR1 as a valid predictor of response to ET. Understanding the mechanisms of acquired resistance to ET can impact therapeutic strategies to overcome the effects of mutant genes responsible for ET failure. Analysis of ESR1 mutations conferring resistance to ET has already been demonstrated in patients with ER+ advanced stage BC (31, 33). Furthermore, *in vitro* studies have shown that ESR1 mutations are likely to be acquired because of ET deprivation (34). However, ESR1 mutations are rare in endocrine therapy-naive ER+/HER2− BC, and the frequency is even lower if an AI has not been administered in the adjuvant setting: 3%–6% (17, 35). In contrast, studies that enrolled patients after first-line AI therapy found that approximately 30% of patients have ESR1mutated (17, 21, 36–41). In the current meta-analysis, the incidence rate of ESR1 was 23%, which was consistent with the results of previous trials.

To date, screening for ESR1 in ER+ BC is not considered the standard of care; tumor tissue sampling remains the standard method for addressing tumor biology, despite issues in terms of acquisition and utility; tissue biopsies are invasive and do not have potential complications, and sample preservation may hamper the use of tumor tissue for cancer sequencing (42). Intra/inter-tumor heterogeneity, mostly observed in advanced cancers, is also a major limitation of tumor biopsy (13, 43). This heterogeneity is partially attributed to dynamic genetic changes that occur after therapeutic selective pressure (44). Therefore, tissue biopsy may not be the most appropriate method for mutational analysis of metastatic BC, especially when looking for rare point mutations in a background of wild-type sequences, as in the case of ESR1.

Liquid biopsy is a rapid, cost-effective, and noninvasive technique, capable of capturing molecular heterogeneity during disease evolution and potentially overcoming the aforementioned issues (44, 45). Cell-free DNA (cfDNA) is a potential surrogate for the entire tumor genome (46). The cfDNA fragments provide a representative reflection of genomic alterations of the original tumor because cfDNA fragments are derived from all tumor sites in a patient’s body circulation (45, 47). Acquired resistance to endocrine therapy prior to disease progression could also be monitored by longitudinally analysis of ESR1 mutations (33). ctDNA analyses are highly sensitive because DNA is abundant in most advanced malignancies, allowing the successful tracking of ESR1 mutations (44). Therefore, liquid biopsy is widely available and easier to perform than standard tumor biopsies (48). Recent improvements in PCR techniques for analyzing cfDNA provide a potential alternative to tumor biopsies, provide information on tumor genetic alterations, and have been used as diagnostic, prognostic, or even predictive tools (49). Our results showed no statistical difference in ESR1 incidence for plasma-tissue comparison (21% vs. 26%; P = 0.34), in accordance with the results of previous reports (21, 23, 29, 30, 32).

At present, ddPCR represents a low-cost and effective technique that has been recently commercialized to detect and quantify small amounts of genetic material (50, 51). ddPCR is a potential alternative to next-generation sequencing (NGS); however, it is only suitable for testing known mutations. Recently, PCR-based digital investigations have been coupled with techniques that use NGS to enumerate rare mutant variants in complex DNA mixtures (52). Both techniques support the screening and clinical validity of genomic alterations in ctDNA as a ‘liquid biopsy’ in breast cancer, including ESR1 mutants (53, 54). ddPCR is particularly useful for the detection of rare mutant DNA sequences in large quantities of background wild-type sequences. Our results showed no statistical difference in ESR1 incidence between the ddPCR-NGS comparisons (26% vs. 19%; P = 0.15).

Although the analysis of cfDNA is a truly growing field, liquid biopsy is not yet routinely used in clinical practice to decode the tumor genome, despite the fact that acquiring plasma samples is more accessible and minimally invasive compared to tissue samples. Furthermore, when comparing the cost-effectiveness of ddPCR and NGS, there was no clear winner. It is generally accepted that ddPCR is a low-cost, time-saving, and effective method for genomic analyses (55, 56). Moreover, ddPCR is designed highly sensitive detection of hotspot mutations, making it more suitable for the detection of low concentrations of cfDNA in plasma samples. NGS relies on different reagents but is capable of testing multiple samples for multiple genes simultaneously. This process is, of course, more time-consuming (7–10 days) and less cost-effective (55, 56). However, assuming a fair number of samples to be tested in routine practice, ddPCR may be a cost-effective and time-sparing method, on the condition that hotspot mutations of interest are known, as is the case for ESR1. In this case, ddPCR may require as little as half the cost of NGS. In our opinion, the analysis of liquid biopsy using ddPCR is the most favorable combination for ESR1 testing in terms of sample feasibility, time, and cost. **Table 3** shows the potential advantages of liquid biopsy compared to tissue biopsy.

**Table 3:** The comparison of liquid versus tissue samples for DNA analysis.

	Liquid biopsy	Tissue biopsy	In favor of
**Invasive method**	Minimally	More invasive Might require surgical intervention	Liquid biopsy
**Longitudinal monitoring**	Easy	Difficult	Liquid biopsy
**Accessibility**	Easy	More challenging	Liquid biopsy
**Tumor heterogeneity**	Covered	Minimally covered	Liquid biopsy
**Tumor material**	Less	More	Tissue biopsy
**DNA concentrations**	Low	High	Tissue biopsy
**Complications**	Low morbidity: Phlebitis	Higher morbidity: More risk of bleeding, infection and surgical complications	Liquid biopsy
**Cost**	ddPCR: Low NGS: High	ddPCR: Moderate NGS: Moderate	*Liquid biopsy if ddPCR
**Sample processing and preservation**	Easy: EDTA-tubes Centrifugation Freezing	Difficult & time-consuming More expensive Formalin fixation Paraffin embedding Large storage rooms	Liquid biopsy

*Assuming routine practice with fair amount of samples.

To our knowledge, this is the largest meta-analysis to carry out a comparative analysis between liquid and tissue biopsies, and between ddPCR and NGS. The results of this review show no significant difference in prevalence of ESR1 mutation detected with liquid biopsy or tissue biopsy. Different studies show a large variability in the prevalence of ESR1 mutations (11% to 55%). The wide range in the incidence rates of ESR1 mutations could be attributed to heterogeneity in the study populations and inter-laboratory findings. A recent review on the progress in detecting ESR1 mutations based on liquid biopsy and different sequencing technologies in ER+ MBC also highlights its potential clinical impacts and prospects in accordance with these conclusions (57).

According to the hypothesis of this review, there was a risk of selection bias because the selected patients had progressive and recurrent BC. Furthermore, meta-analyses on their own may suffer from several sources of bias in individuals and across studies. First, not all trials lead to publication, which induces publication bias for positive findings, and the language of the original publication might have resulted in a selection bias. For some research questions, only a small number of studies were included in the meta-analysis. The quality of the studies varied. Due to the broad scope of our research questions, not only randomized controlled trials, but also case–control and uncontrolled cohort trials were eligible for inclusion in the review. Confounding and baseline differences may be more pronounced in non-randomized or uncontrolled studies than in randomized controlled trials. Furthermore, paired tissue plasma samples were available for only 5.2% of samples. Taken together, these limitations discourage the difficulty of obtaining evidence that plasma is non-inferior to tissue, since both have been measured in different patients and in different studies; solid proof for such a conclusion could only be derived from a large-scale prospective study comparing tissue and plasma samples from the same patients.

This meta-analysis demonstrates that ESR1 mutations are found at high frequency in liquid biopsies of ER+ recurrent/metastasized BC and could be tracked relatively simply and inexpensively using both ddPCR and NGS technologies. Both technologies are equally effective for the identification of ESR1 mutations in tissue and plasma samples; however, ddPCR is inexpensive. Regular ESR1 mutation analysis is needed during endocrine treatment before disease recurrence or progression. The incorporation of cfDNA-based ESR1 analysis is the current challenge for clinicians to ensure that ESR1 testing can be integrated into routine clinical care; however, widespread diagnostic application requires for rigorous studies to demonstrate not only clinical validity but also clinical utility. Recent data from a small cohort of patients suggest that liquid biopsy can reveal the presence of minimal residual disease several years before the appearance of clinically detectable metastatic disease, demonstrating that comprehensive liquid biopsy analysis provides important information for the therapeutic management of breast cancer patients (58). However, the clinical utility of ESR1 analysis as an early predictor needs to be proven in a randomized prospective clinical setting to guide therapeutic decisions on liquid biopsy analysis and on established endpoints (59). Ongoing trials in this setting, such as the SERENA 6, have already addressed the efficacy and safety of switching the ET partner of first-line CDK4/6i therapy at the earliest time point when ESR1m is detected in ctDNA, and before clinical disease progression (60).

In conclusion, the present pooled meta-analysis only provides additional evidence that liquid biopsies can replace tumor tissue biopsies in molecular screening programs for ESR1 mutations in a potentially easier and cost-effective approach. However, the key question of whether changing therapy based on ESR1 mutations before radiologic progression will improve long-term disease control and OS compared to therapy changes based on radiologic progression is yet to be answered.

## Author contributions

Study design, analysis, and interpretation of data: ON. Acquisition of data, technical laboratory support: GB. Statistical analysis: ON, KP, and WT. Study supervision: MH, KP, and WT. ON wrote the manuscript. The final manuscript was edited, reviewed and approved by MH, KP, GB, and WT.
